# From text to e-text: perceptions of medical, dental and allied students about e-learning

**DOI:** 10.1016/j.heliyon.2022.e12157

**Published:** 2022-12-08

**Authors:** Ayesha Fahim, Sadia Rana, Irsam Haider, Varda Jalil, Saira Atif, Sadia Shakeel, Ahsan Sethi

**Affiliations:** a University College of Dentistry, University of Lahore, Lahore, Pakistan; b Sharif Medical & Dental College, University of Health Sciences, Lahore, Pakistan; c Avicenna Medical and Dental College, University of Health Sciences, Lahore, Pakistan; d Institute of Dentistry, CMH Medical College, National University of Medical Sciences, Rawalpindi, Pakistan; e Department of Public Health, College of Health Sciences, QU Health, Qatar University, Doha, Qatar

**Keywords:** COVID-19, Pandemic, E-learning, Undergraduate, Medical, Perception

## Abstract

**Objective:**

More than a year ago, Pakistan like the rest of the word, was hit by a global pandemic, due to which students of higher education had to accept the new era and adapt to the electronic learning environment for the very first time. This study aims to analyze the perceptions of medical, dental, and allied health students about e-learning in Pakistan.

**Methods:**

A descriptive, cross-sectional study was conducted throughout the country. A pre-validated, anonymous online questionnaire regarding demographics, past-experience of e-learning, advantages disadvantages of e-learning, and general perception of students towards e-learning was distributed. Descriptive statistics were computed for all demographics. Chi-square test was used to compare the differences of perceptions between pre-clinical year and clinical years students. Chi-square was used to compare overall category-wise positive and negative responses of students. The association between participant demographics and their perception towards e-learning was also calculated using chi square.

**Results:**

A total of 1200 students participated in the study of which 797 (66.4%) were from pre-clinical years and 403 (33.6%) were from clinical years. The major advantage identified by all students was the ‘comfortable environment’ (70%) and ‘technical problems with IT equipment’ was listed as the biggest disadvantage (89%) of e-learning. For preclinical year students, ‘anxiety due to social isolation’ was selected as the biggest issue (p < 0.05) whereas, for clinical year students, it was ‘lack of patient interaction’ (p < 0.05). Overall, 72% of students had a negative perception of e-learning.

**Conclusion:**

After more than a year of online studying, medical and allied students of Pakistan have expressed dissatisfaction towards e-learning. Student-teacher training, student counselling sessions, and innovative techniques need to be introduced to enhance student engagement and reduce pandemic stress.

## Highlights

• Medical and Allied students are not satisfied with e-learning.
• Lack of student engagement and social isolation are major issues.
• Clinical students suffer due to insufficient patient interaction.

## 1 Introduction

In December 2019 the virus COVID-19 hit China and spread throughout the world causing a worldwide pandemic on March 11, 2020 [1]. As of October 2022, there have been about 615 million registered cases of COVID-19 with about 6.5 million confirmed associated deaths in more than 180 countries [2]. The outbreak caused a disruption not only to the medical education, but the entire healthcare system worldwide. The highly contagious virus made it impossible to conduct teaching and training by the traditional face-to-face method [3]. Medical and allied health schools shut down their routine classes because instructors and students alike were at risk of infection. Similarly, the pandemic affected student clerkship rotations as well because medical and allied health teaching involves routine interaction with patients [4]. The institutions had to find an urgent remedy for the calamity at hand.

The consequent implementation of social distancing forced the students to study at home, and institutions had to adopt e-learning for higher education [5]. This shift from face-to-face to e-learning required purpose-built infrastructure, technologically enhanced equipment, sophisticated softwares, online teaching platforms and extensive trainings of teachers and students [6]. In April 2020, nine medical school groups in USA and UK were able to modify and shift their curricula to e-learning in a matter of days by overcoming the time-constraints, increased in institutional support, face-paced technical trainings and positive attitude of all stakeholders [7]. Soon after, other schools in the West adopted e-learning [8, 9]. This sudden adaptation in these countries stems back to the existing e-learning market, which generated US$ 46.7 billion in 2016 in USA. Till 2018, the market grew globally earning US$ 286.62 billion in total [8]. Simultaneously, medical schools in Australia and New Zealand adopted diverse platforms for synchronous and asynchronous teaching of pre-clinical years [10], institutions announced teacher/student policies for e-learning [11] and Imperial College of London conducted the first ever successful online exam for final year medical students [12]. For clinical years, online repository of patients interviews were provided, clinical teachers were teaching online from hospitals and telemedicine technologies were introduced in UK [13]. The student response to evolution in these regions was also positive with high level of satisfaction and learner engagement [14, 15, 16]. Thus, most well-developed countries have endorsed e-learning owing to its immense benefits [17].

With all these advancements underway, not all medical schools in less developed countries could adopt to modern technological ways. Studies conducted in Bangladesh revealed poor student satisfaction towards e-learning [18, 19]. According to literature, Bangladesh lacked preparedness of online classes during pandemic [19]. Similarly, Iranian health educators expressed their concern about their curriculum and content delivery not being suitable during covid lockdown. Medical students in Iran suffered severe mental distress during this time [20, 21]. Several institutes in Saudi Arabia did not adapt a learning management system at the start of lockdown because older faculty members lacked technical skills [22]. Early studies conducted on students during covid-19 revealed several pitfalls of e-learning in medical schools [23]. Similar level of student and teacher unsatisfaction was reported in India and Philippines [24, 25]. In regards to technical skills and preparedness, Pakistan is no different.

Pakistan faced many hurdles during the pandemic. The fragile economy pushed the annual GDP rate from 5.8 to 0.98% in 2020 [26]. With 79% poverty rate and an increase in unemployment, education and healthcare was greatly affected [27]. Before the pandemic, e-learning had limited existence in Pakistan, restricted to a few government introduced tele-courses about social sciences [28]. When the pandemic hit, and students were forced to study online from home, the biggest challenge was the provision of internet, since the remote areas of three large regions; FATA, Balochistan and Gilgit Baltistan did not have any internet supply [27]. Lack of institutional policies, minimal to nontechnical trainings and poor economy led to hit and trial methods of online teaching training in medical, dental and allied schools. Nonetheless, most schools implemented e-learning and have been practicing it for the last one year. Students living in remote areas were equally enforced by the online education system as those living in big cities. It is safe to say that students did not receive uniform teaching experience The effectiveness of e-learning in developing countries is still quite ambiguous and under-researched. Our study is aimed to analyze the perceptions of medical, dental, and allied health students about e-learning in Pakistan.

## 2 Materials and methods

### 2.1 Study design

**Figure 1:**
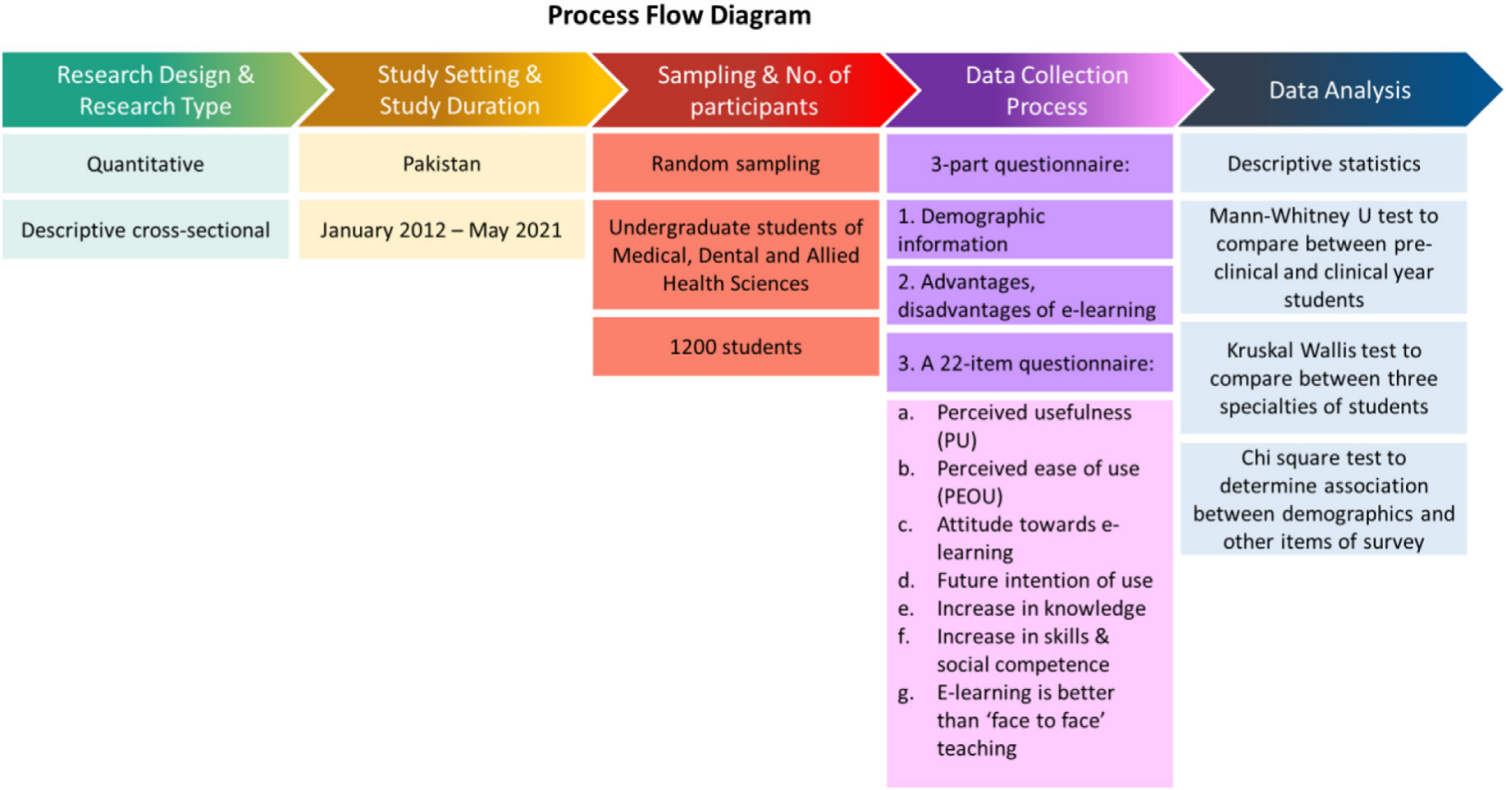
Process flow diagram of research.

A descriptive, cross-sectional study was done from January 2021 till May 2021 to assess the level of acceptance of undergraduate students of Medical (MBBS), Dental (BDS), and Allied Health Sciences (AHS) towards e-learning (Figure 1). The study was conducted in accordance with the declaration of Helsinki and ethical approval was obtained from the parent institution's ethical board (ANDC/RAC/20/04).

### 2.2 Sampling

Purposive, virtual snowball sampling via WhatsApp and Facebook groups was done [29]. In Pakistan, MBBS is five years whereas BDS and bachelor of Allied health sciences are four-year degree programs. Only the students at undergraduate colleges of Pakistan who have shifted to e-learning during the pandemic were included in the study.

### 2.3 Questionnaire

A pre-validated, online, questionnaire designed Mamattah was used for this study [30]. The questionnaire was adapted to fit the medical and allied student context and was validated by five different experts. The modified questionnaire was piloted on 20 students to determine face validity in terms of comprehensiveness and cognitive understanding of the students. The final version was circulated to undergraduate medical, dental, and allied health sciences students in four provinces of Pakistan through WhatsApp, social media groups and email. The official mode of instruction and assessment of medical, dental and allied schools is English, therefore, the questionnaire was not translated in local languages.

The questionnaire consisted of three parts; in the first part, the Initial introduction and objectives of the study were explained followed by the statement of consent. A participant information sheet was provided which stated that student participation is purely voluntary, and it will not affect their assessment or performance in any way. Students were inquired about their demographics and whether they have previous experience of e-learning. Names of students and their institutions were not asked to maintain the anonymity of research and maintain participant confidentiality. In the second part, students were given options regarding the advantages and disadvantages of e-learning. They could choose as many options as they liked. The third part consisted of a 22-item questionnaire, with a 5-point Likert scale; Strongly disagree 1, Somewhat disagree 2, Neutral 3, Somewhat Agree 4, strongly Agree 5. The questions were distributed into seven broad categories: Perceived Usefulness (PU) of e-learning, Perceived ease of use (PEOU), attitude toward e-learning, the future intention of use, increase in knowledge, increase in skills and social competencies, ‘e-learning’ is better than ‘face to face teaching. All items were entered into Google forms (Google LLC) and distributed online to undergraduate students via WhatsApp and email in three waves of invitation: wave 1 (10th January 2021), wave 2 (10th February 2021), and wave 3 (10th March 2021). Data collection was stopped on 10th April 2021 due to time saturation.

### 2.4 Data analysis

All items in the online questionnaire were made mandatory to inhibit missing items [31]. A Mean of 22 items was calculated with scores ranging from 22-110. The Mean score came out to be 55. The respondents who scored more than 55 were considered to have an overall positive perception and those with a score of less than 55 were considered to have an overall negative perception towards e-learning. The items were divided into 7 groups with the following mean values:

a. Perceived usefulness (PU) of e-learning – 4 items (score 4–20, mean 10).
b. Perceived ease of use (PEOU) – 4 items (score 4–20, mean 10).
c. Attitude towards e-learning – 4 items (score 4–20, mean 10).
d. Future intention of use – 4 items (score 4–20, mean 10).
e. Increase in knowledge – 1 item (score 1–5, mean 3).
f. Increase in skills and social competencies – 2 items (score 2–10, mean 5).
g. E-learning is better than ‘face to face’ teaching – 3 items (3–15, mean 8).

A score above the mean score was considered positive response and a score below the mean score was considered negative response. Descriptive statistics (mean, frequencies and percentages) were computed for all demographics. Chi-square test was used to compare the differences of perceptions between pre-clinical year students (Year 1 and 2) and clinical years students (3 till 5), and to compare the results of three specialties of students. Chi-square was also used to compare overall category-wise positive and negative responses of students. Association between participant demographics and their perception towards e-learning was calculated using chi square. All analyses were done using IBM SPSS statistical software, version 24 (IBM Corporation, New York) and Microsoft Excel 2013 (Microsoft Corporation, Redmond, Washington). A *p-value* of less than 0.05 was considered significant.

## 3 Results

### 3.1 Characteristics of respondents

**Figure 2:**
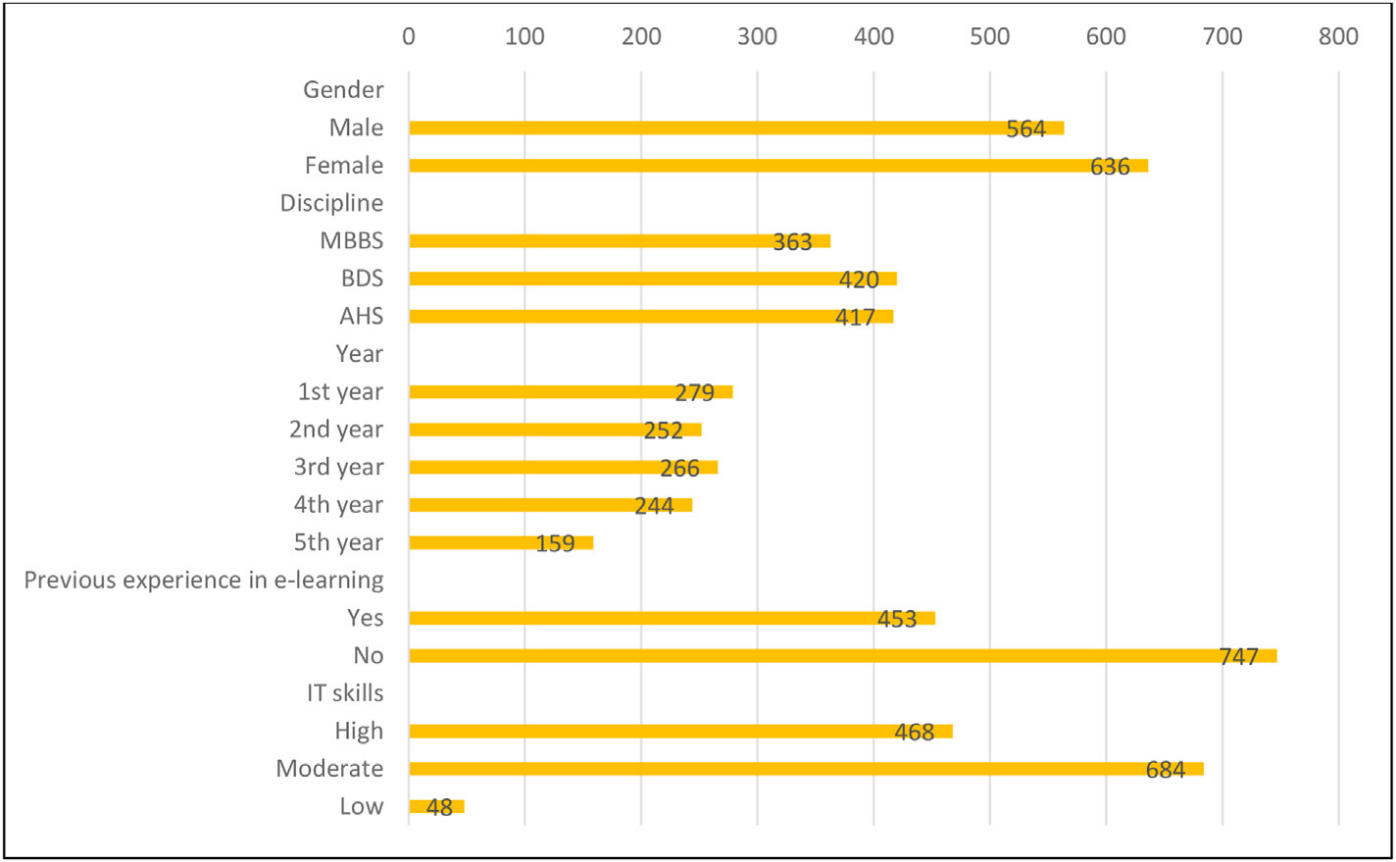
Demographics of students and their past experience of E-learning.

A total of 1200 students participated in the study, out of which 47% (n = 564) were male and 53% (n = 636) were female. Among these, 30.2% (n = 363) were medical (MBBS), 35% (n = 420) were dental (BDS) and 34.7% (n = 417) were allied health (AHS) students. A total of 66.4% (n = 797) were from pre-clinical years and 33.6% (n = 403) were from clinical years. The Cronbach alpha of the questionnaire was 0.81 which denotes ‘good’ reliability. The demographics are presented in Figure 2. The majority of the students considered themselves good at IT (information technology) (∼94%) and only 37.75% of students had previous experience of e-learning.

### 3.2 Advantages and disadvantages of E-learning

The most frequent advantage of e-learning chosen by students was comfortable surroundings (70%), followed by the ability to stay at home (69%) and learning at your own pace (64%). A total of 89% of students listed technical problems with IT equipment as the main disadvantage of e-learning. The second most frequently chosen disadvantage varied for clinical and pre-clinical years. For clinical year students, the lack of interaction with patients was a significantly bigger disadvantage (p < 0.05), whereas, for pre-clinical years, the development of anxiety due to social isolation and lack of interaction with teachers was the second most chosen disadvantage (p < 0.05) (Table 1). The results were non-significant across the three disciplines.

**Table 1:** Advantages and disadvantages of e-learning.

Variables	Pre-clinical years (n = 531)	Clinical years (n = 669)	p-value	MBBS (n = 363)	BDS (n = 420)	AHS (n = 417)	p-value	Total
**Advantages of online learning**								
Access to online material	187	221	0.428	121	140	147	0.800	408 (34%)
Learning at your own pace	350	418	0.219	242	256	270	0.232	768 (64%)
Ability to stay at home	410	418	<0.001	271	290	267	0.005	828 (69%)
Classes interactivity	51	45	0.067	33	29	34	0.526	96 (8%)
Improvement in virtual communication and technical skills	211	149	<0.001	127	122	111	0.034	360 (30%)
Comfortable surrounding	450	390	<0.001	281	259	300	0.001	840 (70%)
**Disadvantages of online learning**								
Reduced interaction with teachers	346∗ (65.2%)	158	<0.001∗	177	178	149	0.001	504 (42%)
Frequent technical problems	510	558	<0.001	346	365	357	<0.001	1068 (89%)
Lack of interaction with patients	40	660∗ (98.6%)	<0.001∗	252	231	217	<0.001	700 (58.3%)
Poor learning conditions at home	136	128	0.007	89	84	91	0.312	264 (22%)
Lack of self-discipline	107	133	0.907	97	80	63	0.000	240 (20%)
Anxiety due to social isolation	478∗ (90%)	194	<0.001∗	209	230	233	0.729	672 (56%)

MBBS: medical students, BDS: dental students, AHS: allied health sciences students.

∗p-value is significant i.e., less than 0.05.

### 3.3 Perception of students towards E-learning

Student perceptions about e-learning were collected (Table 2). About 72% (n = 863) students had an overall negative perception about e-learning. There was no significant difference between the gain in knowledge during face-to-face and e-learning (p > 0.05). However, students had a statistically significant negative perception about the perceived usefulness of e-learning (80% negative), perceived ease of use (70.8% negative), attitude towards e-learning (68% negative), intention for future use (71.8% negative), and increase in skills and social competencies during e-learning (81.2% negative) (p < 0.05). When associated with demographics, the domains perceived usefulness, ease of use, attitude toward e-learning, future intention of use and increased in skills & social competencies were significantly higher in male gender as compared to females (p < 0.05). Similarly, there was significant association of discipline MBBS with overall perception of e-learning, usefulness of e learning, ease of use, increased knowledge and increase in skills and social competencies. There was no significant difference between the responses of pre-clinical and clinical years students (p > 0.05), hence results were not included.

**Table 2:** Overall perception and Category wise responses of students towards E-learning.

Category	Responses	Male n/%		Female n/%		p-value	MBBS n/%		BDS n/%		AHS n/%		p-value
Overall Perception	+ve = 337 (28%)	150	27%	187	29%	0.281	159	44%	119	28%	59	14%	<0.001∗
	−ve = 863 (72%)	414	73%	449	71%		204	56%	301	72%	358	86%	
Perceived Usefulness (PU) of e-learning	+ve = 241 (20%)	131	23%	110	17%	0.010∗	105	29%	90	21%	46	11%	<0.001∗
	−ve = 959 (80%)	433	77%	526	83%		258	71%	330	79%	371	89%	
Perceived ease of use (PEOU)	+ve = 350 (29.1%)	200	35%	150	24%	<0.001∗	140	39%	105	25%	105	25%	<0.001∗
	−ve = 850 (70.8%)	364	65%	486	76%		223	61%	315	75%	312	75%	
Attitude toward e-learning	+ve = 384 (32%)	200	35%	184	29%	0.015∗	126	35%	125	30%	133	32%	0.337
	−ve = 816 (68%)	364	65%	452	71%		237	65%	295	70%	284	68%	
Future intention of use	+ve = 339 (28.3%)	180	32%	159	25%	0.007∗	110	30%	121	29%	108	26%	0.376
	−ve = 861 (71.8%)	384	68%	477	75%		253	70%	299	71%	309	74%	
Increase in knowledge	+ve = 628 (52.3%)	290	51%	338	53%	0.551	225	62%	205	49%	198	47%	<0.001∗
	−ve = 572 (47.7%)	274	49%	298	47%		138	38%	215	51%	219	53%	
Increase in skills and social competencies	+ve = 226 (18.8%)	126	22%	100	16%	0.003∗	89	25%	67	16%	70	17%	0.003∗
	−ve = 974 (81.2%)	438	78%	536	84%		274	75%	353	84%	347	83%	
‘E-learning’ is better than ‘face to face’ teaching	+ve = 192 (16%)	92	16%	100	16%	0.781	67	18%	55	13%	70	17%	0.107
	−ve = 1008 (84%)	472	84%	536	84%		296	82%	365	87%	347	83%	

∗p-value is significant i.e., less than 0.05.

## 4 Discussion

In this study, we assessed medical, dental, and allied health students' perceptions about e-learning. It was noticed that only 37.75% of students had previous experience of e-learning, which is different from previously conducted studies in USA and UK where the majority of the students were not new to online education [32, 33, 34]. Online educational courses have been offered to students in the USA, UK, Australia and other well-developed countries even before COVID-19 [35], whereas, only ‘Virtual University of Pakistan’ was offering online teaching programs in Pakistan until a couple of years ago [36]. Our results are comparable with the lesser developed countries like India, Nepal, Jordan and Nigeria where, like Pakistan, e-learning in medical field was entirely new [25]. The participants of this study who claim to have prior e-learning experience were probably those who have attended online classes during COVID lockdown. Owing to limited resources and the level of inhibition towards faculty development programs, several institutions, despite being unprepared, dived into the stream of e-education. This system is not only new for faculty but students as well.

Only 4% of students did not claim to have good command over IT skills, the rest were well versed with technology. Our questionnaire did not specify the IT skills; thus, we expect that participants who are frequent social media users also consider themselves good at IT. It has been previously reported that young people tend to over-report their IT skills [37, 38]. Alternatively, this data may also suggest that students have markedly improved their IT skills in the last year after being exposed to an e-education system [32, 39]. Lack of technical skills is considered as one of the major barriers in acceptance towards online learning [40]. With good to excellent knowledge of IT skills and having to experience e-learning in the past year, we expected that students must have grown accustomed to the new dawn of education. In contrast, it was observed that 72% of students had a negative overall perception towards e-learning. This result links to the disadvantages of online learning selected by participants, however, further studies are required to prove the hypothesis.

The biggest disadvantage selected by participants was frequent technical problems during an online class. This result is in contrast to previously done studies where lack of engagement and improper feedback were considered the greatest disadvantages [41, 42]. An empirical study was conducted in India after launching Massive Open Online Courses (MOOC) in the country during COVID. These courses were believed to have a direct impact on improving educational outcomes and in turn, student satisfaction level [43]. E-learning requires a steady internet connection and continuous electrical supply [44]. Unfortunately, this problem is far from being resolved in a low-income country like Pakistan. Even before COVID, Pakistan was not able to successfully run online programs and the electricity crisis presented a major cause of this [45]. Although different internet providers have invested heavily in Pakistan in an attempt to provide seamless internet connection, but electricity problems especially in rural areas make it difficult to maintain ICT (information communication technology) [46]. Studies have associated frequent technical problems with a high level of anxiety amongst students that lead to poor learning [47]. Literature suggests enhancing the budget for e-education environment for health professionals [48, 49]. Value and cost analysis studies indicate that where a break-even analysis is completed, the e-learning approach was robustly superior to a traditional face-to-face education, allowing lower number of enrolments for a program to reach its break-even point’. While this analysis might not always be an approach adopted by medical schools in developing an online programme, it is suggested as one of the ways in which one might look at the cost of establishing the correct infrastructure not as a barrier but as a potential solution to a barrier [50, 51].

Another major disadvantage pointed out by students was the lack of interaction with teachers and patients. This disadvantage has been observed in several countries including Malaysia [52], Saudi Arabia [53], Jordan [54] and India [55]. About 80% of medical teaching and assessment revolves around patients, lack of which gravely affects educational outcome of a practitioner. These results are consistent with previously conducted studies [32, 56]. All these studies were conducted almost a year ago. Since then, a lot of innovation has been done in teaching clinical years through 3D software, augmented reality, virtual interactive patients, and telemedicine [57], but it seems that students still face the same problems. In China, institutions introduced specialized online clinical courses for students. They adopted the principles of virtual reality using platforms like ilab.-x.com, live broadcasts, recorded broadcasts, MOOC and video-conferencing to enhance student engagement and satisfaction [58]. For clinical assessment, online OSPE and OSCE are being conducted [59]. To effectively conduct online clinical teaching and assessment in Pakistan, institutions require high end software trainings of faculty which can be arranged through collaborations between the tech companies and medical institutions.

The strongest advantage of e-learning as perceived by medical, dental, and allied students is the availability of a comfortable environment, followed by the opportunity to learn at their own pace. These results are consistent with previous studies [60]. Student concentration increases substantially with a favorable environment [61]. Some studies contradict this result. A study conducted on Dutch students concluded that although student motivation decreased during stay-at-home study, their academic performance did not decrease [62]. These results support the notion that self-directed and instructor-directed e-learning allows learners to manage their time independently and effectively. Several studies advocate self-directed learning as being more effective than face-to-face learning [63].

Where self-directed e-learning has its advantage, it causes social isolation in students sitting at home [64]. This is reflected in our results as well where most of the pre-clinical years students have chosen ‘anxiety due to social isolation’ as a major disadvantage of e-learning. Similar reports have been found in Bangladesh [65], Philippines [25], Sri Lanka [66], Saudi Arabia [67] and Hong Kong [68]. Immense research has been conducted in the last few years, adding a variety of innovations in health professionals education [69]. Various researchers have presented ‘tips’ for online student engagement [70], on how to conduct clinical sessions [71] and to cope with Pandemic stress disorder [72]. Faculty training sessions are required to train teachers on student engagement. Similarly, students should be counseled regularly, and their academic progress must be monitored continuously to note signs of anxiety and lack of interest.

To our knowledge, this is the first study that aims to analyze perceptions of students after one year of online education. Although students in Pakistan have been subjected to e-learning for the past year, the level of satisfaction amongst students is still quite poor. Students do not believe that e-learning can enhance their clinical or social skills. They do not perceive its usefulness and do not find it easy to use. To our surprise, students did not find any significant difference in the gain of knowledge between e-learning and face-to-face learning. We recommend that longitudinal studies must be conducted to assess the improvement of student perception. Future qualitative studies can help us better understand students’ emotional responses and ways of improving them in health sector education. Our study has few limitations. We did not address potential issues like time management, student-teacher training on online educational platforms, effective feedback on learning, isolation anxiety and perception of all stakeholders including teaching faculty, administrators and parents of students.

## 5 Conclusion

Our findings indicate that even though the undergraduate medical, dental and allied health students considered e-learning to have advantages like comfortable surroundings and the ability to study at home, the disadvantages outweigh them. Majority of the students of Pakistan were not satisfied with e-learning even after being exposed to it for more than a year. Pakistan, being a middle to low-income country, cannot be expected to provide stable and fast internet connections throughout the country especially in rural areas within the next 5 years. Keeping all limitations in mind, the government and institutional stakeholders should work together to cope with the inevitable future of e-learning in medical field.

## Declarations

### Author contribution statement

Ayesha Fahim; Sadia Rana; Irsam Haider: Conceived and designed the experiments; Contributed reagents, materials, analysis tools or data; Wrote the paper.

Varda Jalil; Saira Atif: Performed the experiments; Contributed reagents, materials, analysis tools or data; Wrote the paper.

Sadia Shakeel: Analyzed and interpreted the data; Contributed reagents, materials, analysis tools or data; Wrote the paper.

Ahsan Sethi: Conceived and designed the experiments; Analyzed and interpreted the data; Contributed reagents, materials, analysis tools or data; Wrote the paper.

### Funding statement

This research did not receive any specific grant from funding agencies in the public, commercial, or not-for-profit sectors.

### Data availability statement

Data will be made available on request.

### Declaration of interest's statement

The authors declare no competing interests.

### Additional information

No additional information is available for this paper.

## Appendix A Supplementary data

questionnaire.pdf questionnaire.pdf
