# Supplementary material for: From text to e-text: perceptions of medical, dental and allied students about e-learning
Source: Heliyon. 2022 Dec 8;8(12):e12157. doi: 10.1016/j.heliyon.2022.e12157 (PMC9758397; doi:10.1016/j.heliyon.2022.e12157)
Supplement: questionnaire.pdf [file mmc1.pdf]

Factors that Influence Students' Acceptance towards E-learning

Dear Respondent,

Greetings to you!

Due to the COVID-19 pandemic, we are all in a situation where we have no choice but to implement, cope and adopt to e-learning platforms to carry on with our classes. In this study, we have chosen to investigate the main factors which influence Pakistani Medical, Dental and allied health sciences undergrad and post grad students' acceptance towards e-learning and we truly appreciate your kind participation in this survey to contribute to our study!

*This survey should take you within 5 minutes to complete.
* We ensure you the anonymity and you may opt not to to participate in this study if you wish so.

*Required

1. Do you consent to this study? *

Mark only one oval.

- Yes
- No

2. Specify your field of study *

Mark only one oval.

- medical
- dental
- allied health sciences

3. Specify the type of study *

Mark only one oval.

- undergraduate
- postgraduate

4. Please specify your gender *

Mark only one oval.

- Male
- Female

5. What is your year of study?

Mark only one oval.

- first year
- second year
- third year
- fourth year
- fifth year

6. Have you ever experienced online learning / e-learning? *

Mark only one oval.

- Yes
- No

7. How would you rat your IT / computer operating skills? *

Mark only one oval.

- High
- Moderate
- Low

Part 2

8. In your opinion, What are the advantages of online / e-learning? *

Tick all that apply.

- [ ] Access to online material
- [ ] Learning at your own pace
- [ ] Ability to stay at home
- [ ] Interactivity in classes
- [ ] Improvement in virtual communication and technical skills
- [ ] Comfortable surrounding
- [ ] Other: ______________________

9. In your opinion, what are the disadvantages of online / e-learning? *

Tick all that apply.

- [ ] Reduced interaction with teachers
- [ ] Frequent technical problems
- [ ] Lack of interaction with patients
- [ ] Poor learning conditions at home
- [ ] Lack of self-discipline
- [ ] Anxiety due to social isolation
- [ ] Other: ______________________

Perceived Usefulness (PU)

10. Studying through e-learning mode provides the flexibility to study at the time convenient to the learner *

Mark only one oval.

	1	2	3	4	5	
strongly disagree	◯	◯	◯	◯	◯	strongly agree

11. Using E-learning would enhance my effectiveness in learning *

Mark only one oval.

	1	2	3	4	5	
strongly disagree	◯	◯	◯	◯	◯	strongly agree

12. Using E-learning would improve my course performance *

Mark only one oval.

	1	2	3	4	5	
strongly disagree	◯	◯	◯	◯	◯	strongly agree

13. E-learning can enable people to study, irrespective of where they are located in the world. *

Mark only one oval.

	1	2	3	4	5	
strongly disagree	◯	◯	◯	◯	◯	strongly agree

Perceived Ease Of Use PEOU

14. My interaction with E-learning is clear and understandable *

Mark only one oval.

	1	2	3	4	5	
Strongly disagree	◯	◯	◯	◯	◯	Strongly agree

15. It will be easy for me to find information through E-learning. *

Mark only one oval.

	1	2	3	4	5	
Strongly disagree	◯	◯	◯	◯	◯	Strongly agree

16. I believe e-learning platforms are user friendly *

Mark only one oval.

	1	2	3	4	5	
Strongly disagree	◯	◯	◯	◯	◯	Strongly agree

17. I can easily download resource material on my cellphone / laptop *

Mark only one oval.

	1	2	3	4	5	
Strongly disagree	◯	◯	◯	◯	◯	Strongly agree

Attitude towards E learning

18. I like the idea of e-learning *

Mark only one oval.

	1	2	3	4	5	
strongly disagree	◯	◯	◯	◯	◯	strongly agree

19. I think e-learning is an innovation and must be encouraged with on campus learning *

Mark only one oval.

	1	2	3	4	5	
strongly disagree	⬭	⬭	⬭	⬭	⬭	strongly agree

20. I do not find any advantage of E learning over face to face learning *

Mark only one oval.

	1	2	3	4	5	
strongly disagree	⬭	⬭	⬭	⬭	⬭	strongly agree

21. Using E-learning is a foolish idea *

Mark only one oval.

	1	2	3	4	5	
strongly disagree	⬭	⬭	⬭	⬭	⬭	strongly agree

Future Intention Of Use

22. I intend to use E-learning during the whole semester

Mark only one oval.

	1	2	3	4	5	
Strongly disagree	◯	◯	◯	◯	◯	strongly agree

23. I will return to E-learning often, but not all the time

Mark only one oval.

	1	2	3	4	5	
Strongly disagree	◯	◯	◯	◯	◯	strongly agree

24. I like combination of online learning and face-to face learning (hybrid learning

Mark only one oval.

	1	2	3	4	5	
Strongly disagree	◯	◯	◯	◯	◯	strongly agree

25. I prefer fully-online learning to face-to-face

Mark only one oval.

	1	2	3	4	5	
Strongly disagree	◯	◯	◯	◯	◯	strongly agree

Increase in Knowledge

26. My knowledge of subject has increased because of e-learning

Mark only one oval.

	1	2	3	4	5	
Strongly disagree	◯	◯	◯	◯	◯	Strongly agree

Increase in skills and social competencies

27. There can be interactive communication of students with teachers when participating in e-learning

Mark only one oval.

	1	2	3	4	5	
Strongly disagree	◯	◯	◯	◯	◯	Strongly agree

28. There can be interactive communication among students when participating in e-learning

Mark only one oval.

	1	2	3	4	5	
Strongly disagree	◯	◯	◯	◯	◯	Strongly agree

E-learning is better than ‘face to face’ teaching

29. I prefer E-learning over face-to-face learning *

Mark only one oval.

	1	2	3	4	5	
strongly disagree	◯	◯	◯	◯	◯	Strongly agree

30. I am comfortable with the fully face-to-face than online learning

Mark only one oval.

	1	2	3	4	5	
Strongly disagree	◯	◯	◯	◯	◯	strongly agree

31. I am satisfied with e-learning in my institute

Mark only one oval.

	1	2	3	4	5	
Strongly disagree	◯	◯	◯	◯	◯	strongly agree

This content is neither created nor endorsed by Google.

Google Forms
